# The Association between Human Papillomavirus Infection and Smoking, Age, Gender in Lung Cancer Patients: A Meta-Analysis

**Published:** 2019-01

**Authors:** Rui ZHANG, Ling CHEN, Ya-Deng CUI, Ge LI

**Affiliations:** 1. School of Public Health and Management, Research Center for Medicine and Social Development, Chongqing Medical University, Chongqing, 400016, China; 2. Collaborative Innovation Center of Social Risk Governance in Health, Chongqing Medical University, Chongqing, 400016, China; 3. The Center of Experimental Teaching Management, Chongqing Medical University, Chongqing, 401331, China

**Keywords:** Human papillomavirus, Smoking, Meta-analysis, Gender, Age

## Abstract

**Background:**

The aim of our study was to identify the association between Human papillomavirus (HPV) positive rate and smoking in lung cancer (LC) patients. Meanwhile, to analyze differences among gender, age differences on HPV infection rate in LC patients.

**Methods:**

We performed a systematic literature search through PubMed, Wan Fang, China National Knowledge Infrastructure (CNKI), MEDLINE, EMBASE (OVID), and Web of Science databases from 1991–2017, and we searched these keywords such as “lung cancer”, “HPV”, “smoking”, and “human papillomavirus”. Review Manager 5.3 software was used to analyze. An estimate of the odds ratio (OR) with 95% confidence intervals (CI) was calculated.

**Results:**

In China, a statistical significance was observed between HPV positive rate and smoking in LC patients (OR=2.34, 95%CI: 1.76–3.09, *P*<0.001; I^2^ =25%). However, after stratified by region, no significance was observed in other regions, gender, and age.

**Conclusion:**

HPV infections are associated with smoking in LC patients. The association between HPV infection and smoking in LC patients may relate to different regions. There were no differences between gender and age among HPV infection rates in LC patients. To identify the etiology of smoking, HPV, and LC, a further experimental research needs to be conducted.

## Introduction

Lung cancer (LC) is the most common cause of morbidity and mortality around the world. According to the statistical data (1), there were 1.8 million new cases and 1.6 million deaths in 2012. Now the pathogenesis of LC is inconsistent. Although smoking is one of the major factors in the development of LC, about 25% of patients with LC are non-smoker. Hence, the occurrence of LC has many potential risk factors, such as the occupational exposure of asbestos and radon, environmental pollution, biological carcinogenic factors and so on. With the detection of human papillomavirus in LC, people have paid attention to the viral infection which is the carcinogenic factor of LC.

Human papillomavirus (HPV), a small and naked deoxyribonucleic acid (DNA) virus, consisting of double-stranded circular DNA, is believed to be an important factor contributing to the etiology of certain benign and malignant lesions in humans. HPV infections are associated with up to 35% of oropharyngeal cancers (2). In recent years, with the rapid development of molecular biology, increasing evidence suggests that HPV may play an important role in the development of LC. Since 1980, HPV infection may relate to the development of LC. People began to pay attention to the association between LC and HPV infection and its possible carcinogenic mechanism (3). However, the evidence on effect of HPV on the development of LC is still inconsistent. HPV infection increased LC risk (4), and HPV16/18 infection increased the risk of lung squamous cell carcinoma. This meta-analysis suggested that HPV infection is an important factor in the prognosis of LC. However, this study did not report the association between HPV and clinical features in LC patients, such as smoking status, age, region, gender and so on. Hence, we aimed to study the association between HPV and smoking, gender, and age in LC patients.

## Methods

### Search strategy

We performed a systematic literature study with multiple strategies: 1) electronic database searches, such as PubMed, Wan Fang, China National Knowledge Infrastructure (CNKI), MEDLINE, EMBASE (OVID), and Web of Science, and keywords such as “lung cancer,” “HPV,” “smoking,” and “human papillomavirus,” were used; 2) request for articles to researchers; 3) review of reference sections of articles obtained from searches. Studies matched with the selection criteria and available from 1991–2017, were included in the analysis. This meta-analysis was performed in accordance with the guidelines of Preferred Reporting Items for Systematic Reviews and Meta-Analyses (PRISMA) (5).

### Study selection and inclusion criteria

Studies were selected if they met the following criteria: 1) they were case-control, cross-sectional or cohort studies comparing HPV infection in lung tissue among LC patients and non-cancer controls; 2) research involved smoking; 3) they provided information needed to calculate odds ratio (OR) with 95% confidence intervals. We excluded studies in which the subject population were not LC patients. Duplicate studies, reviews, local reports, conference abstracts, and presentations were excluded. When an overlap of patients was found in several studies, only the study with the largest sample size and detailed information was included. Two co-authors (Rui Z, Ling C) independently extracted relevant studies following the inclusion criteria (6). Disagreements were resolved through discussion in a panel meeting (6). The characteristics of the records included in the meta-analysis are shown in Table 1.

**Table 1:** Characteristics of the 19 eligible studies in this meta-analysis

***Author***	***Year***	***Region***	***HPV(+)***							
			***Ag <50***	***Age≥50***	***Age<60***	***Age≥60***	***Smoking***	***No Smoking***	***Male***	***Female***
Zhou et al. ( 16 )	2015	China			8/16	22/47	20/38	10/25	19/43	11/20
Zhou et al. ( 15 )	2015	China			6/15	19/45	18/43	7/17	19/46	6/14
Xiong et al. ( 14 )	2016	China			3/43	4/40	6/44	1/39	6/53	1/30
Zhang et al. ( 13 )	2012	China			8/23	9/20	9/20	8/23	13/30	4/13
Chen et al. (12)	2005	China					43/85	18/41	42/91	19/35
Liu et al.(11)	2007	China	6/21	29/68			26/64	9/25	28/69	7/20
Zhang et al. (24)	2016	China			3/21	5/30	6/28	2/23	5/29	3/22
S.A.Nadji et al. (8)	2007	Iran			5/12	28/114	28/108	5/21	27/104	6/25
E.Sarchianaki et al. (9)	2014	Greece					8/38	0/7	17/91	2/9
Yang et al. (10)	2006	China					12/33	11/40	14/39	9/34
Zhou et al. (23)	2014	China					15/22	2/14	15/26	2/12
Jiang er al. (22)	2005	China					18/38	3/22	17/43	4/17
Yuan et al. (19)	2006	China	6/17	25/59			26/50	5/26	24/59	7/17
Zhen et al. (21)	2015	China	2/6	15/37			12/22	4/21	12/30	4/13
Huang et al. (20)	2006	China	8/15	11/29			15/26	4/18	13/34	6/10
Wang et al. (18)	2005	China	6/12	12/30			15/26	3/16	14/32	4/14
Kong et al. (25)	2009	China					18/33	2/14	15/35	5/12
Yan et al. (17)	2009	China					31/57	4/11		
E.Argyri et al. (26)	2017	Greece					2/66	0/1	1/58	1/9

### Data extraction

Screening of the title and abstract was performed independently in the first step, and disagreement was resolved by discussion. Full-text review was retrieved and then detailed evaluation was followed (6). All data extraction were conducted independently and checked by two authors, disagreements being resolved by discussion.

### Statistical analysis

Odds ratios (ORs) with corresponding 95% CIs were calculated if there were sufficient data. Heterogeneity of these studies was evaluated using the *P-*value and the I^2^ statistic (7). If I^2^<50%, a fixed-effect model was used to evaluate inter-study heterogeneity; otherwise, a random-effect model was used. All statistical analysis was carried out with the use of Review Manager 5.3 (Cochrane). Moreover, *P*-values less than 0.05 were considered statistically significant. All statistical tests were two-sided.

### Eligible studies

**Fig. 1:**
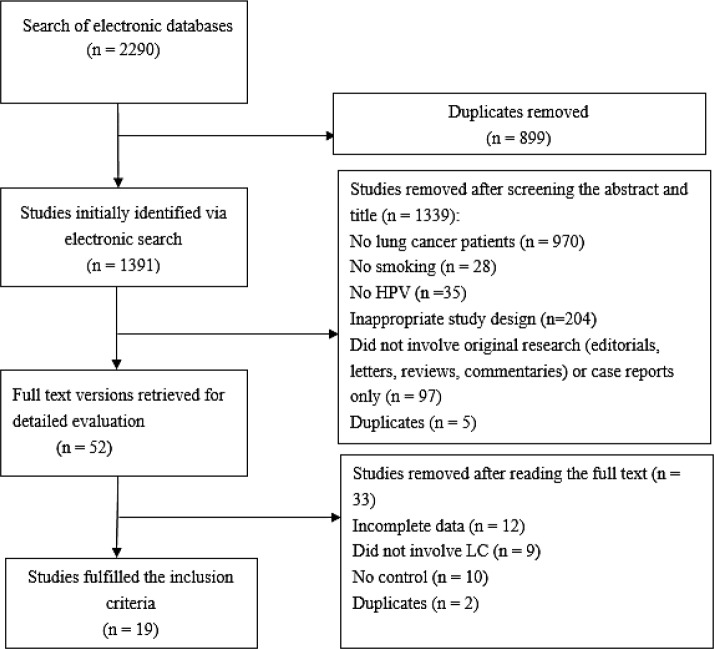
Search strategy

This study of the electronic databases revealed 2290 studies, of which 899 overlapped among different search categories. The search strategy in Fig. 1 as the QUOROM statement flowchart in which the detailed procedure of reference identification along with information regarding exclusion criteria applied at different stages of the selection is described. After screening the title and abstract of the 1391 unique references, 1339 were excluded, and 52 studies were required for further assessment. After screening full-text reviews, 33 studies excluded, while only 19 (8–26) articles fulfilled the predefined inclusion criteria and were selected to be involved in the analysis, eligible studies in this meta-analysis.

We identified 19 studies around the world, 16 studies of which were from China, and 3 studies from Greece and Iran. Studies contained 1245 samples. All studies have reported prevalence of HPV, patients’ demography, gender differences in HPV positive rate, HPV detection methods, HPV positive rate in smoking LC patients, and other significant information.

### Ethics approval

The study’s protocol and data collection procedure were approved by the Institute of Public Health and Management. No individual data are used; only group data are reported. Thus, consent is not applicable.

## Results

### Association between HPV positive rate and smoking status of LC patients

**Fig. 2:**
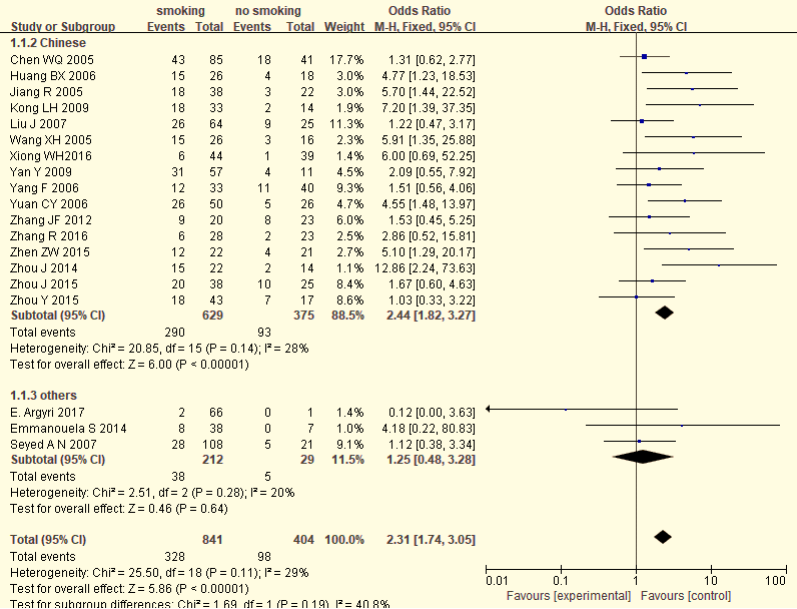
The relationship between HPV positive rate and smoking in LC patients

HPV positive rate in smoking LC patients was higher than that in non-smoking LC patients (39.00% VS. 24.26%). Fig. 2 shows a forest plot of the overall association between HPV positive rate and smoking status of LC patients. A statistical significance was observed between HPV positive rate and smoking status of LC patients (OR=2.31, 95%CI: 1.74–3.05, *P*<0.001; I^2^ =29%). In addition, after stratified by region, significance was also detected in 16 Chinese studies (OR=2.44, 95%CI: 1.82–3.27, *P*<0.001; I^2^ =28%). However, no significance was showed in 3 other studies (OR=1.25, 95%CI: 0.48–3.28, *P*=0.64; I^2^ =20%).

### Gender differences of HPV positive rate in patients with LC

**Fig. 3:**
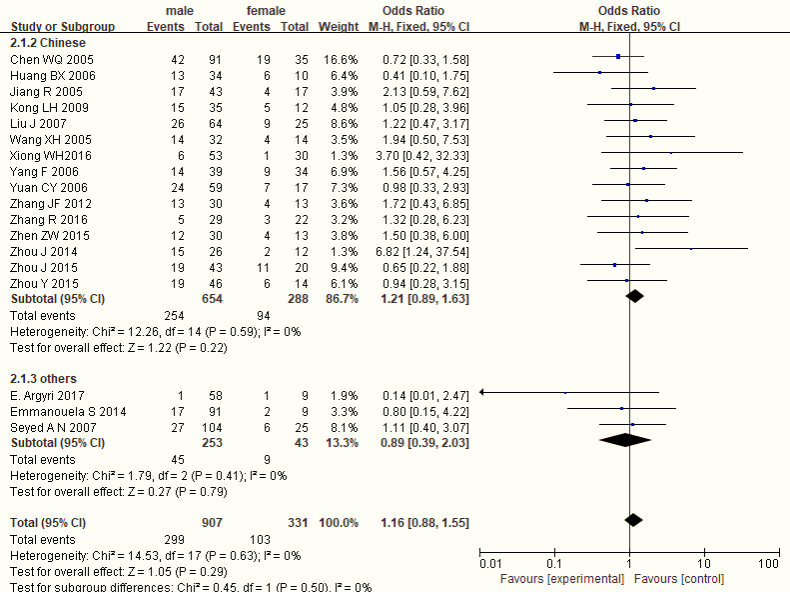
Gender differences in HPV positive rate in LC patients

Fig. 3 shows a forest plot of the gender differences of HPV positive rate in LC patients, stratified by region. No statistical significance was observed between gender and HPV in LC patients (OR=1.16, 95%CI: 0.88–1.55, P=0.29; I^2^ =0%), also in different region.

### Age differences of HPV positive rate in patients with LC

**Fig. 4:**
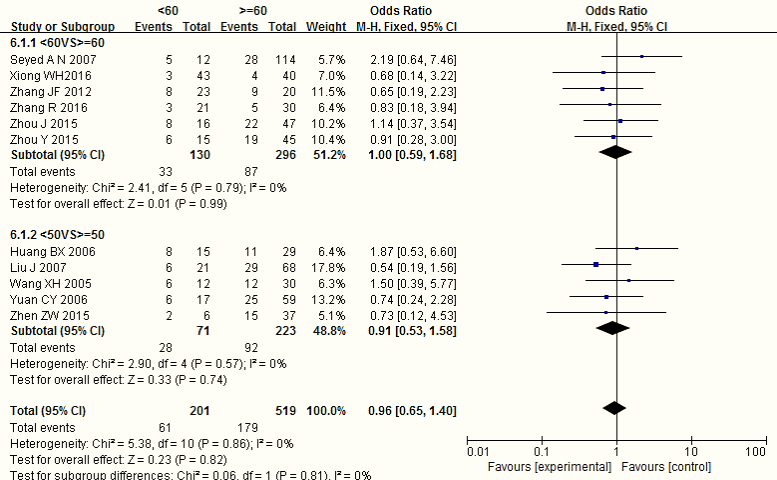
Age differences in HPV positive rate in patients with LC

Fig. 4 shows a forest plot of the age differences of HPV positive rate in LC patients, stratified by different age group. No statistical significance was observed between age and HPV in LC patients (OR=0.96, 95%CI: 0.65–1.40, *P*=0.86; I^2^ =0%).

### Sensitivity analysis

To assess the sensitivity of this meta-analysis, we sequentially removed individual studies from it. The pooled ORs had good stability, and statistical significance was found by fix-effect model. The results of this meta-analysis are reliable. Moreover, funnel plot indicates that no significant publication bias exists in this review.

## Discussion

HPV is thought to be a high risk of lung cancer. The HPV infection rate in smoker was higher than that in non-smoker LC patients. However, HPV infection rate was not related to smoking in the development of LC. The association between smoking and HPV infection and whether these two factors exert a synergistic effect on the development of LC are still in the dispute.

Now, HPV infection was closely related to smoking in the occurrence of LC. Smoking could directly cause HPV infection, since smoking can decrease Langhans cell which is antigen-presenting cell in epithelial tissue, then lead to immune deficiency, which is helpful to the activation of HPV and the persistence of infection (27). All HPV infection cases were moderate smokers in LC patients (28). Both HPV infection and P53 protein expression were associated with smoking, and smoking and HPV infection may have a synergic effect on the development of LC (19). However, there was no significant difference between HPV infection and smoking in LC patients (11). HPV was thought to be an environmental pollution factor, which may lead to airway injury, and eventually, lead to LC. There was no significant difference between smoking and non-smoking in HPV infection rate in LC patients, which indicated that smoking may be an independent carcinogenic factor in the development of LC, and no synergic effect with HPV infection (10, 29). Our study showed that HPV positive infection are associated with smoking in LC patients, smoking can lead to HPV infection; HPV infection and smoking have a synergic effect on the development of LC. Meanwhile, HPV is the risk factor of LC, this is consistent with previous studies.

In the study of LC and HPV infection, we found that not only smoking status but also age, and gender are significant clinical features. There were regional differences in HPV infection in non-smokers with LC that East Asia was higher than Europe (30). The HPV infection rate was similar in non-smoker and quit-smoking populations in Asia, while in Europe non-smoker was higher that quit-smoking people. The HPV infection rate of non-smoker was 68.7% in Taiwan, 60% in Korea, 23.8% in China and 12.4% in Japan (30). HPV infection is associated with different region, which was inconsistent with our study. We conducted a stratified analysis to study the regional differences. After stratified by region, a statistical significance was detected in Chinese, not in others, which suggested that in China, HPV infections are associated with smoking in LC patients. The association between HPV infection and smoking in LC patients may be related to different regions. The lack of statistical significance in other countries may be due only 2 other studies included.

The incidence rate was higher in male and in the elder. However, age and gender were always ignored as the mixed factors in the study of HPV infection and LC. Few studies reported the association between age, gender and HPV infection in LC patients. There was no association between HPV infection and gender in LC patients (31). Our study made two forest plots for gender and age differences in HPV positive rate in LC patients. There were no significant gender and age differences in HPV positive rate in LC patients, which suggested that there was no association among age, gender and HPV infection in LC patients (Figs. 3,4).

Consequently, HPV infection may relate to smoking and region in LC patients, but not to gender and age. The detection rate may alter with different detection methods in different study. For lack of sample size, there were some limitations in our study. Therefore, a large sample of study was needed to investigate the synergistic effect on smoking and HPV infection in LC, also mechanism needed. So our study suggested that smoking may be a risk factor of HPV infection in LC patients, and it promotes the development of LC. Moreover, region is associated with HPV infection rate in smoking LC patients, age but gender are not.

Some limitations exist in our meta-analysis, 16 studies from China, while 2 other studies, which may lead to regional bias. HPV gene type may have an association with smoking in LC. However there was only one study involves HPV gene type, therefore, we are unable to analyze the association between HPV gene type and smoking in LC. Although our study suggested that HPV infections are associated with smoking in LC patients, we do not know the certain etiology of smoking, HPV, and LC. Smoking increased HPV infection, which in turn caused LC, however the exact mechanism required further experimental research.

## Conclusion

The forest plot shows that HPV infections are associated with smoking in LC patients. Unfortunately, most of our involved studies are from China, which may lead to regional bias. After stratified by age and gender, no statistical differences were observed in our study. Hence, smoking may be a risk factor of HPV infection in LC patients, gender and age may not relate to HPV infection in LC patients.

## Ethical consideration

Ethical issues (Including plagiarism, informed consent, misconduct, data fabrication and/or falsification, double publication and/or submission, redundancy, etc.) have been completely observed by the authors.
